# 7-Acylamino-3H-1,2-benzoxathiepine 2,2-dioxides as new isoform-selective carbonic anhydrase IX and XII inhibitors

**DOI:** 10.1080/14756366.2020.1722658

**Published:** 2020-02-21

**Authors:** Aleksandrs Pustenko, Alessio Nocentini, Anastasija Balašova, Mikhail Krasavin, Raivis Žalubovskis, Claudiu T. Supuran

**Affiliations:** a Latvian Institute of Organic Synthesis, Riga, Latvia; b Institute of Technology of Organic Chemistry, Faculty of Materials Science and Applied Chemistry, Riga Technical University, Riga, Latvia; c Dipartimento Neurofarba, Sezione di Scienze Farmaceutiche e Nutraceutiche, Università degli Studi di Firenze, Florence, Italy; d Department of Chemistry, Saint Petersburg State University, Saint Petersburg, Russian Federation

**Keywords:** Carbonic anhydrase, transmembrane isoforms, sulfocoumarin, homosulfocoumarin, isoform-selective inhibitor

## Abstract

A series of 3H-1,2-benzoxathiepine 2,2-dioxides incorporating 7-acylamino moieties were obtained by an original procedure starting from 5-nitrosalicylaldehyde, which was treated with propenylsulfonyl chloride followed by Wittig reaction of the bis-olefin intermediate. The new derivatives, belonging to the homosulfocoumarin chemotype, were assayed as inhibitors of the zinc metalloenzyme carbonic anhydrase (CA, EC 4.2.1.1). Four pharmacologically relevant human (h) isoforms were investigated, the cytosolic hCA I and II and the transmembrane, tumour-associated hCA IX and XII. No relevant inhibition of hCA I and II was observed, whereas some of the new derivatives were effective, low nanomolar hCA IX/XII inhibitors, making them of interest for investigations in situations in which the activity of these isoforms is overexpressed, such as hypoxic tumours, arthritis or cerebral ischaemia.

## 1. Introduction

Sulfocoumarins (1,2-benzoxathiine 2,2-dioxides) and homosulfocoumarins (3H-1,2-benzoxathiepine 2,2-dioxides)^1–5^ are among the most investigated new classes of carbonic anhydrase (CA, EC 4.2.1.1) inhibitors, which have been designed considering the structurally similar coumarins^6–8^ as lead molecules. Indeed, Cas are widely spread enzymes in organisms of all types, from simple to complex ones^9–15^, and are involved in crucial physiological processes, among which carbon fixation in diatoms and other marine organisms in which several genetic families of such metalloenzymes were reported^9^. In protozoans, Cas are involved in biosynthetic reactions^9^ whereas in bacteria, where at least three genetic families were described (α-, β-, and γ-Cas) these enzymes play crucial roles related both to metabolism but also virulence and survival in various niches^10^. In vertebrates, including humans, a high number of different CA isoforms belonging to the α-CA class were described^11^^,^^12^, which by hydrating CO_2_ to a weak base (bicarbonate) and a strong acid (hydronium ions), are involved in a multitude of processes, starting with pH regulation and ending with metabolism^13^^,^^14^. As thus, Cas are drug targets for decades, with their inhibitors having pharmacological applications in a multitude of fields^11–16^. The primary sulphonamides were discovered as CA inhibitors (CAIs) in the ‘40 s, and most of the drugs that were launched in the next decades as diuretics, antiepileptics, or antiglaucoma agents belonged to this class of compounds or to their isosteres such as the sulfamates and sulfamides^11^. An important drawback of such first generation CA inhibitors (CAIs) was their lack of isoform selectivity, considering the fact that in humans at least 12 catalytically active and three acatalytic isoforms are present^11^^,^^12^. However, the new generation CAIs to which coumarins and sulfocoumarins belong, show significant isoform-selective inhibition profiles, as demonstrated in a considerable number of studies^1–8^. This is principally due to the fact that these compounds possess a distinct inhibition mechanism compared to the sulphonamides, which coordinate to the zinc ion from the CA active site as anions^11^^,^^12^. In fact, coumarins and sulfocoumarins act as prodrug inhibitors, undergoing an active site mediated hydrolysis, which leads to the formation of 2-hydroxy-cinnamic acids in the case of the coumarins, and ethane-sulphonates in the case of the sulfocoumarins, which subsequently bind in different active site regions, different of those where the classical sulphonamide CAIs bind^1–8^. As shown by X-ray crystallography, the hydrolysed coumarins occlude the entrance of the CA active site cavity^6^, whereas the sulfocoumarins bind deeper within the active site, but still do not coordinate to the metal ion. Instead, the formed sulphonates anchor to the zinc-coordinated water molecule, as shown again by means of X-ray crystallographic techniques^2^. As these regions of the CA active site are the most variable ones, a straightforward explanation of the isoform selectivity of these new generation CAIs was furnished by using a combination of crystallographic and kinetic studies, which also allowed the development of compounds showing a higher degree of selectivity^15^^,^^16^. This allowed for the development of inhibitors useful for new pharmacological applications such as antitumor/antimetastatic compounds^13^, CAIs useful for the management of arthritis^17^, neuropathic pain^18^, and cerebral ischaemia^19^.

Considering our interest in designing non-sulphonamide CAIs with various potential applications, we report here a new series of homosulfocoumarins and their inhibitory profiles against the major human (h) CA isoforms, hCA I, II, IX, and XII, involved in many pathologies, including cancer.

## 2. Experimental part

### 2.1. Chemistry

Reagents, starting materials/intermediates **1–7** and solvents were obtained from commercial sources (Sigma-Aldrich, St. Louis, MO) and used as received. Anhydrous CH_2_Cl_2_ and toluene were obtained by passing commercially available solvents through activated alumina columns. Thin-layer chromatography was performed on silica gel, spots were visualised with UV light (254 and 365 nm). Melting points were determined on an OptiMelt automated melting point system. IR spectra were recorded on Shimadzu FTIR IR Prestige-21 spectrometer. NMR spectra were recorded on Bruker Avance Neo (400 MHz) spectrometer with chemical shifts values (δ) in ppm relative to TMS using the residual DMSO-d_6_ signal (^1^H 2.50; ^13 ^C 39.52) or CDCl_3_ signal (^1^H 7.26; ^13 ^C 77.16) as an internal standard. High-resolution mass spectra (HRMS) were recorded on a mass spectrometer with a Q-TOF micro mass analyser using the ESI technique.

#### General procedure for synthesis of acyl compound 8–17

To a solution of amino derivative **7** (1.0 eq.) in dry CH_2_Cl_2_ (20 ml per mmol of compound **7**) at 0 °C appropriate acyl chloride (1.1 eq.) and Net_3_ (1.1 eq.) were added. The resulting mixture was stirred at room temperature under an argon atmosphere for 2 h. Water was added (20 ml per mmol of compound 7). Layers were separated, water layer was washed with EtOAc (2 × 40 ml). Combined organic layers were washed with brine, dried over anh. Na_2_SO_4_, filtered, evaporated. The crude solids were recrystallised form EtOAc/petrol ether mixture to afford product.

##### *N*-(2,2-Dioxido-3*H*-1,2-benzoxathiepin-7-yl)acetamide (8)


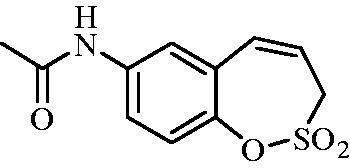


Compound **8a** was prepared according to the general procedure from amino derivative **7** (150 mg; 0.71 mmol), acetyl chloride (56 µL; 0.78 mmol) and Et_3_N (110 µL; 0.78 mmol) as white solid (127 mg; 70%). Mp 164–165 °C.

IR (film, cm^−1^) *ν*_max_= 3276 (N–H), 1670 (C = O), 1370 (S = O), 1361 (S = O), 1166 (S = O), 1162 (S = O);

^1^H NMR (400 MHz, DMSO-d_6_) *δ* = 2.06 (s, 3H), 4.37–4.41 (m, 2H), 5.96–5.6.04 (m, 1H), 6.89 (d, 1H, *J* = 11.3 Hz), 7.28 (d, 1H, *J* = 8.9 Hz), 7.58 (dd, 1H, *J* = 8.9, 2.5 Hz), 7.69 (d, 1H, *J* = 2.5 Hz), 10.16 (s, 1H) ppm.

^13^C NMR (100 MHz, DMSO-d_6_) *δ* = 24.0, 51.0, 120.6, 120.8, 122.7, 128.4, 131.5, 138.0, 142.2, 168.6 ppm. 

HRMS (ESI) [M + H]^+^: *m*/*z* calcd for (C_11_H_12_NO_4_S) 254.0487. Found 254.0498.

##### *N*-(2,2-Dioxido-3*H*-1,2-benzoxathiepin-7-yl)benzamide (9)


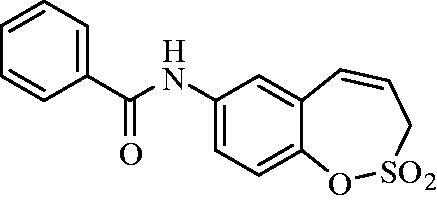


Compound **9** was prepared according to the general procedure from amino derivative **7** (150 mg; 0.71 mmol), benzoyl chloride (90 µL; 0.78 mmol) and Et_3_N (110 µL; 0.78 mmol) as white solid (162 mg; 72%). Mp 174–175 °C.

IR (film, cm^−1^) *ν*_max_= 3289 (N–H), 1652 (C = O), 1370 (S = O), 1363 (S = O), 1163 (S = O);

^1^H NMR (400 MHz, DMSO-d_6_) *δ* = 4.43 (dd, 2H, *J* = 6.0, 0.9 Hz), 5.99–6.06 (m, 1H), 6.93 (d, 1H, *J* = 11.2 Hz), 7.35 (d, 1H, *J* = 8.8 Hz), 7.52–7.58 (m, 2H), 7.59–7.64 (m, 1H), 7.82 (dd, 1H, *J* = 8.8, 2.5 Hz), 7.91 (d, 1H, *J* = 2.5 Hz), 7.94–7.99 (m, 2H), 10.46 (s, 1H) ppm.

^13^C NMR (100 MHz, DMSO-d_6_) *δ* = 51.1, 120.9, 122.0, 122.1, 122.6, 127.7, 128.3, 128.5, 131.4, 131.8, 134.6, 137.9, 142.7, 165.7 ppm

HRMS (ESI) [M + H]^+^: *m*/*z* calcd for (C_16_H_14_NO_4_S) 316.0644. Found 316.0654.

##### *N*-(2,2-Dioxido-3*H*-1,2-benzoxathiepin-7-yl)-4-methy benzamide (10)


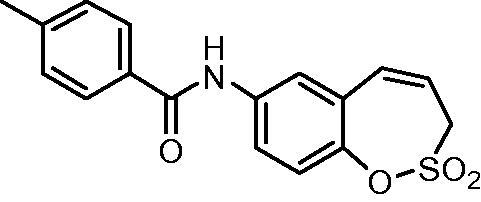


Compound 10 was prepared according to the general procedure from amino derivative **7** (150 mg; 0.71 mmol), 4-methylbenzoyl chloride (103 µL; 0.78 mmol) and Et_3_N (110 µL; 0.78 mmol) as white crystals (170 mg; 73%). Mp 197–198 °C.

IR (film, cm^−1^) *ν*_max_ = 3324 (N–H), 1646 (C = O), 1378 (S = O), 1363 (S = O), 1177 (S = O), 1169 (S = O);

^1^H NMR (400 MHz, DMSO-d_6_) *δ* = 2.39 (s, 3H), 4.41–4.45 (m, 2H), 5.99–6.06 (m, 1H), 6.92 (d, 1H, *J* = 11.2 Hz), 7.32–7.37 (m, 3H), 7.82 (dd, 1H, *J* = 8.9, 2.6 Hz), 7.86–7.92 (m, 3H), 10.37 (s, 1H) ppm

^13^C NMR (100 MHz, DMSO-d_6_) *δ* = 21.0, 51.1, 120.8, 121.9, 122.1, 122.6, 127.7, 128.3, 129.0, 131.4, 131.6, 138.0, 141.9, 142.6, 165.5 ppm

HRMS (ESI) [M + H]^+^: *m*/*z* calcd for (C_17_H_16_NO_4_S) 330.0800. Found 330.0815.

##### *N*-(2,2-Dioxido-3*H*-1,2-benzoxathiepin-7-yl)4-bromobenzamide (11)


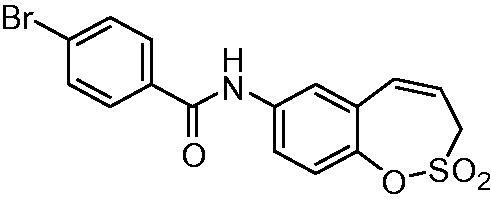


Compound **11** was prepared according to the general procedure from amino derivative **7** (150 mg; 0.71 mmol), 4-bromobenzoyl chloride (171 mg; 0.78 mmol) and Et_3_N (110 µL; 0.78 mmol) as white solid (166 mg; 59%). Mp 185–186 °C.

IR (film, cm^−1^) *ν*_max_= 3260 (N–H), 1653 (C = O), 1375 (S = O), 1363 (S = O), 1167 (S = O);

^1^H NMR (400 MHz, DMSO-d_6_) *δ* = 4.42–4.46 (m, 2H), 5.99–6.06 (m, 1H), 6.92 (d, 1H, *J* = 11.3 Hz), 7.35 (d, 1H, *J* = 8.8 Hz), 7.74–7.83 (m, 3H), 7.88–7.94 (m, 3H), 10.52 (s, 1H) ppm

^13^C NMR (100 MHz, DMSO-d_6_) *δ* = 51.2, 120.9, 122.0, 122.2, 122.7, 125.6, 128.3, 129.8, 131.4, 131.5, 133.6, 137.7, 142.8, 164.7 ppm

HRMS (ESI) [M + H]^+^: *m*/*z* calcd for (C_16_H_13_BrNO_4_S) 393.9749. Found 393.9736.

##### *N*-(2,2-Dioxido-3*H*-1,2-benzoxathiepin-7-yl)-2-iodobenzamide (12)


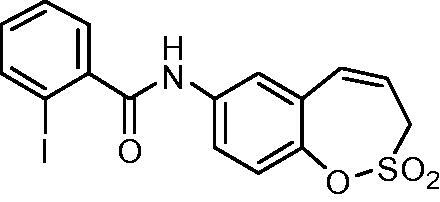


Compound **12** was prepared according to the general procedure from amino derivative **7** (150 mg; 0.71 mmol), 2-iodobenzoyl chloride (208 mg; 0.78 mmol) and Et_3_N (110 µL; 0.78 mmol) as white solid (276 mg; 88%). Mp 188–189 °C.

IR (film, cm^−1^) *ν*_max_= 3240 (N–H), 1641 (C = O), 1374 (S = O), 1362 (S = O), 1156 (S = O);

^1^H NMR (400 MHz, DMSO-d_6_) *δ* = 4.41–4.45 (m, 2H), 6.00–6.08 (m, 1H), 6.94 (d, 1H, *J* = 11.2 Hz), 7.22–7.28 (m, 1H), 7.36 (d, 1H, *J* = 8.8 Hz), 7.47–7.55 (m, 2H), 7.72 (dd, 1H, *J* = 8.8, 2.5 Hz), 7.87 (d, 1H, *J* = 2.5 Hz), 7.9–7.97 (m, 1H), 10.67 (s, 1H) ppm

^13^C NMR (100 MHz, DMSO-d_6_) *δ* = 51.0, 93.6, 121.0, 121.2, 121.3, 122.9, 128.1, 128.2, 128.5, 131.2, 131.5, 137.7, 139.1, 142.7, 142.8, 167.7 ppm

HRMS (ESI) [M + H]^+^: *m*/*z* calcd for (C_16_H_13_INO_4_S) 441.9610 Found 441.9609.

##### *N*-(2,2-Dioxido-3*H*-1,2-benzoxathiepin-7-yl)-2-bromobenzamide (13)


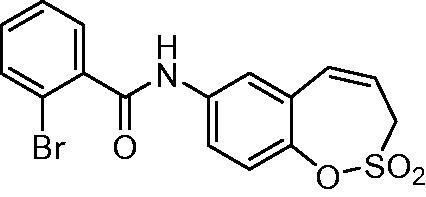


Compound **13** was prepared according to the general procedure from amino derivative **7** (150 mg; 0.71 mmol), 2-bromobenzoyl chloride (102 µL; 0.78 mmol) and Et_3_N (110 µL; 0.78 mmol) as white solid (230 mg; 82%). Mp 177–178 °C.

IR (film, cm^−1^) *ν*_max_= 3288 (N–H), 1653 (C = O), 1371 (S = O), 1176 (S = O), 1156 (S = O);

^1^H NMR (400 MHz, DMSO-d_6_) *δ* = 4.43 (dd, 2H, *J *=* *6.0, 0.9 Hz), 6.00–6.07 (m, 1H), 6.94 (d, 1H, *J* = 11.2 Hz), 7.36 (d, 1H, *J* = 8.9 Hz), 7.41–7.47 (m, 1H), 7.51 (dt, 1H, *J* = 7.4, 1.1 Hz), 7.55–7.59 (m, 1H), 7.69–7.76 (m, 2H), 7.87 (d, 1H, *J* = 2.6 Hz), 10.73 (s, 1H) ppm

^13^C NMR (100 MHz, DMSO-d_6_) *δ* = 51.0, 118.9, 121.1, 121.2, 122.9, 127.8, 128.6, 128.9, 131.4, 131.5, 132.8, 137.6, 138.8, 142.8, 166.0 ppm

HRMS (ESI) [M + H]^+^: *m*/*z* calcd for (C_16_H_13_BrNO_4_S) 393.9749 Found 393.9766.

##### *N*-(2,2-Dioxido-3*H*-1,2-benzoxathiepin-7-yl)-2-fluorobenzamide (14)


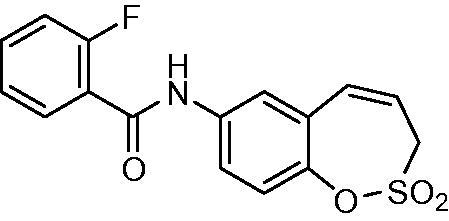


Compound **14** was prepared according to the general procedure from amino derivative **7** (150 mg; 0.71 mmol), 2-fluorobenzoyl chloride (93 µL; 0.78 mmol) and Et_3_N (110 µL; 0.78 mmol) as white solid (188 mg; 79%). Mp 173–174 °C.

IR (film, cm^−1^) *ν*_max_= 1671 (C = O), 1371 (S = O), 1365 (S = O), 1164 (S = O) 1156 (S = O);

^1^H NMR (400 MHz, DMSO-d_6_) *δ* = 4.41–4.45 (m, 2H), 6.00–6.07 (m, 1H), 6.93 (d, 1H, *J* = 11.2 Hz), 7.32–7.40 (m, 3H), 7.56–7.63 (m, 1H), 7.65–7.71 (m, 1H), 7.74 (dd, 1H, *J* = 8.8, 2.5 Hz), 7.86 (d, 1H, *J* = 2.5 Hz), 10.65 (s, 1H) ppm

1. ^13^C NMR (100 MHz, DMSO-d_6_) *δ* = 51.1, 116.2 (d, *J* = 21.7 Hz), 121.0, 121.4, 121.5, 122.8, 124.6 (d, *J* = 5.5 Hz), 124.7 (d, *J* = 6.3 Hz), 128.5, 129.9 (d, *J* = 2.6 Hz), 131.4, 132.8 (d, *J* = 8.5 Hz), 137.5, 142.8, 159.9 (d, *J* = 249 Hz), 163.0 ppm
2. HRMS (ESI) [M + H]^+^: *m*/*z* calcd for (C_16_H_13_FNO_4_S) 334.0549 Found 334.0554.

##### *N*-(2,2-Dioxido-3*H*-1,2-benzoxathiepin-7-yl)-2-(trifluoromethyl)benzamide (15)


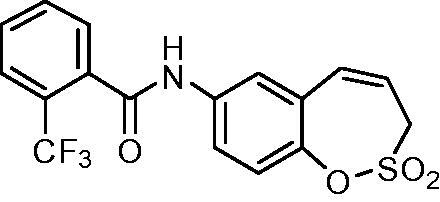


Compound **15** was prepared according to the general procedure from amino derivative **7** (150 mg; 0.71 mmol), 2-(trifluoromethyl)benzoyl chloride (115 µL; 0.78 mmol) and Et_3_N (110 µL; 0.78 mmol) as white solid (236 mg; 87%). Mp 192–193 °C.

IR (film, cm^−1^) *ν*_max_= 3195 (N–H), 1666 (C = O), 1377 (S = O), 1316 (S = O), 1164 (S = O);

^1^H NMR (400 MHz, DMSO-d_6_) *δ* = 4.42–4.45 (m, 2H), 6.00–6.08 (m, 1H), 6.94 (d, 1H, *J* = 11.2 Hz), 7.36 (d, 1H, *J* = 8.8 Hz), 7.67–7.76 (m, 3H), 7.78–7.89 (m, 3H), 10.81 (s, 1H) ppm

^13^C NMR (100 MHz, DMSO-d_6_) *δ* = 51.0, 121.1, 121.3, 122.9, 123.8 (q, *J* = 274 Hz), 125.8 (q, *J* = 31.2 Hz), 126.4 (q, *J* = 4.6 Hz), 128.5, 128.6, 130.3, 131.4, 132.7, 135.8 (q, *J* = 2.3 Hz), 137.6, 142.8, 165.8 ppm

HRMS (ESI) [M + H]^+^: *m*/*z* calcd for (C_17_H_13_NO_4_ F_3_S) 384.0517 Found 384.0519.

##### *N*-(2,2-Dioxido-3*H*-1,2-benzoxathiepin-7-yl)thiophene-2-carboxamide (16)


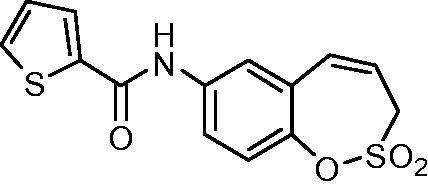


Compound **16** was prepared according to the general procedure from amino derivative **7** (150 mg; 0.71 mmol), 2-thiophenecarbonyl chloride (84 µL; 0.78 mmol) and Et_3_N (110 µL; 0.78 mmol) as white solid (185 mg; 81%). Mp 162–163 °C.

IR (film, cm^−1^) *ν*_max_= 3357 (N–H), 1648 (C = O), 1372 (S = O), 1356 (S = O), 1178 (S = O), 1165 (S = O);

^1^H NMR (400 MHz, DMSO-d_6_) *δ* = 4.44 (dd, 2H, *J* = 6.0, 1.1 Hz), 5.99–6.06 (m, 1H), 6.92 (d, 1H, *J* = 11.2 Hz), 7.23–7.26 (m, 1H), 7.35 (d, 1H, *J* = 8.8 Hz), 7.78 (dd, 1H, *J* = 8.8, 2.6 Hz), 7.84 (d, 1H, *J* = 2.6 Hz), 7.88 (dd, 1H, *J* = 5.0, 1.1 Hz), 8.04 (dd, 1H, *J* = 3.8, 1.1 Hz), 10.43 (s, 1H) ppm

^13^C NMR (100 MHz, DMSO-d_6_) *δ* = 51.2, 120.9, 121.9, 122.1, 122.7, 128.2, 128.4, 129.5, 131.3, 132.3, 137.5, 139.5, 142.7, 160.0 ppm

HRMS (ESI) [M + H]^+^: *m*/*z* calcd for (C_14_H_12_NO_4_S_2_) 322.0208 Found 322.0221.

##### *N*-(2,2-Dioxido-3*H*-1,2-benzoxathiepin-7-yl)furan-2-carboxamide (17)


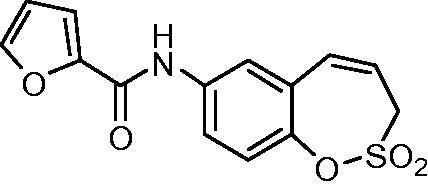


Compound **17** was prepared according to the general procedure from amino derivative **7** (150 mg; 0.71 mmol), 2-furoyl chloride (84 µL; 0.78 mmol) and Et_3_N (110 µL; 0.78 mmol) as white solid (185 mg; 81%). Mp 162–163 °C.

IR (film, cm^−1^) *ν*_max_= 3299 (N–H), 1663 (C = O), 1367 (S = O), 1363 (S = O), 1165 (S = O), 1158 (S = O);

^1^H NMR (400 MHz, DMSO-d_6_) *δ* = 4.41–4.45 (m, 2H), 5.98–6.06 (m, 1H), 6.70–6.74 (m, 1H), 6.90 (d, 1H, *J* = 11.2 Hz), 7.32–7.38 (m, 2H), 7.80 (dd, 1H, *J* = 8.8, 2.6 Hz), 7.87 (d, 1H, *J* = 2.6 Hz), 7.95–7.97 (m, 1H), 10.41 (s, 1H) ppm

^13^C NMR (100 MHz, DMSO-d_6_) *δ* = 51.2, 112.3, 115.2, 120.9, 122.0, 122.2, 122.6, 128.3, 131.4, 137.3, 142.7, 146.0, 147.2, 156.3 ppm

HRMS (ESI) [M + H]^+^: *m*/*z* calcd for (C_14_H_12_NO_5_S) 306.0436 Found 306.0463.

### 2.2. CA inhibitory assay

An applied photophysics stopped-flow instrument has been used for assaying the CA catalysed CO_2_ hydration activity^20^. Phenol red (at a concentration of 0.2 mM) was used as indicator, working at the absorbance maximum of 557 nm, with 20 mM Hepes (pH 7.5) as buffer and 20 mM Na_2_SO_4_ (for maintaining constant the ionic strength), following the initial rates of the CA-catalysed CO_2_ hydration reaction for a period of 10 − 100 s. The CO_2_ concentrations ranged from 1.7 to 17 mM for the determination of the kinetic parameters and inhibition constants. For each inhibitor, at least six traces of the initial 5 − 10% of the reaction have been used for determining the initial velocity. The uncatalysed rates were determined in the same manner and subtracted from the total observed rates. Stock solutions of inhibitor (0.1 mM) were prepared in distilled − deionised water, and dilutions up to 0.01 nM were done thereafter with the assay buffer. Inhibitor and enzyme solutions were preincubated together for 6 h at room temperature prior to assay in order to allow for the formation of the E − I complex. The inhibition constants were obtained by nonlinear least-squares methods using PRISM 3 and the Cheng − Prusoff equation, as reported earlier^21–23^, and represent the mean from at least three different determinations. All CA isoforms were recombinant ones obtained in-house as reported earlier^21^^,^^24^.

## 3. Results and discussion

### 3.1. Chemistry

**Scheme 1.**
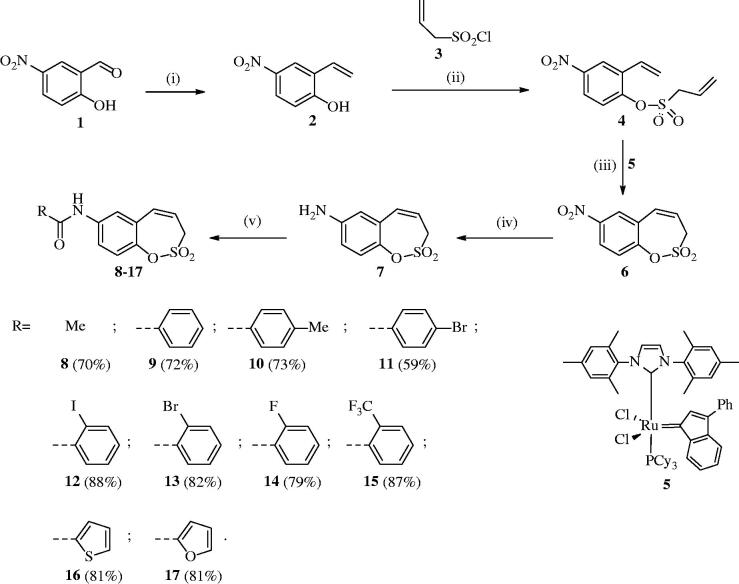
Reagents and conditions: (i) MePPh_3_Br, tBuOK, THF, RT, 18 h, 65%; (ii) Net_3_, CH_2_Cl_2_, 0 °C to RT, 4 h, 57%; (iii) **5**, toluene, 70 °C, 4 h, 96%; (iv) Fe, AcOH, EtOH, H_2_O, 75 °C, 1 h, 98%; (v) RCOCl, Net_3_, CH_2_Cl_2_, 0 °C to RT, 4 h.

Starting from the benzaldehyde derivative **1**, the synthesis of the key intermediate **7** was reported earlier by our groups^1^. Briefly, the synthesis of 7-amino-3H-1,2-benzoxathiepine 2,2-dioxide (**7**) was started with a Wittig reaction in which 5-nitro-salicylic aldehyde **1** was converted to the corresponding mono-olefin **2** in 65% yield (Scheme 1). Treatment of compound **2** with allyl sulphonyl chloride (**3**) provided the bisolefin **4** in 65% yield. In the next step, the olefin metathesis reaction with Ru-catalyst **5** was employed, leading to the conversion of compound **4** to 7-nitro-3H-1,2-benzoxathiepine 2,2-dioxide **6** in 96% yield. The nitro derivative **6** was thereafter reduced with iron in acidic medium to the corresponding amine **7** in nearly quantitative yield (98%). The key intermediate **7** was subsequently reacted with a series of acyl chlorides to afford the desired compounds **8–17** in good to excellent yields (see Experimental for details). The nature of moieties R was chosen in such a way to assure chemical diversity. Apart R = Me in compound **8**, the remaining derivatives **9–17** incorporated aromatic or heterocyclic moieties, such as phenyl, 2- or 4-substituted phenyls, thienyl and furyl. We found out in previous papers^1–3^ that aryl or hetaryl moieties on the sulfocoumarin, homosulfocoumarin or coumarin ring^6^ systems lead to compounds with an effective inhibition profile against CA isoforms of pharmacologic interest, such as the tumour-associated ones CA IX and XII.

### 3.2. Carbonic anhydrase inhibition

The obtained homosulfocoumarins **8–17** were investigated for their CA inhibitory properties by using a stopped-flow CO_2_ hydrase assay^20^ and four human CA isoforms (hCA I, II, IX, and XII) known to be drug targets^1^ (Table 1).

**Table 1.** Inhibition data of human CA isoforms CA I, II, IX and XII with 3H-1,2-benzoxathiepines 2,2-dioxide **8–17** using acetazolamide (**AAZ**) as a standard drug.
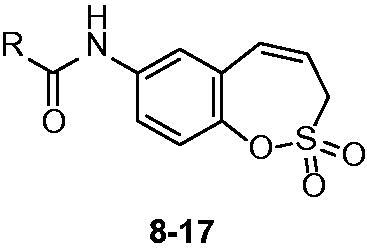

Cmpd	R	K_I_ (nM)^a,b^			
		hCA I	hCA II	hCA IX	hCA XII
**8**	CH_3_	>100 µM	>100 µM	61.8	162.5
**9**	C_6_H_5_	>100 µM	>100 µM	208.6	370.1
**10**	4-CH_3_-C_6_H_4_	>100 µM	>100 µM	83.0	309.3
**11**	4-Br-C_6_H_4_	>100 µM	>100 µM	353.3	140.7
**12**	2-I-C_6_H_4_	>100 µM	>100 µM	45.4	643.7
**13**	2-Br-C_6_H_4_	>100 µM	>100 µM	66.8	96.2
**14**	2-F-C_6_H_4_	>100 µM	>100 µM	74.6	40.3
**15**	2-CF_3_-C_6_H_4_	>100 µM	>100 µM	19.7	8.7
**16**	thien-2-yl	>100 µM	>100 µM	177.5	73.2
**17**	furan-2-yl	>100 µM	>100 µM	210.1	134.4
**AAZ**	–	250	12	25	5.7

^a^Mean from three different assays, by a stopped flow technique (errors were in the range of ±5–10% of the reported values).

^b^Incubation time 6 h.

As seen from data of Table 1, derivatives **8–17** did not significantly inhibit the cytosolic isoforms hCA I and II, similar to other homosulfocoumarins, sulfocouamrins or coumarins investigated earlier^1–8^. On the other hand, the transmembrane, tumour-associated isoforms hCA IX and XII were inhibited by all these compounds in the nanomolar range. For hCA IX the K_I_s were in the range of 19.7–353.3 nM whereas for hCA XII in the range of 8.7–643.7 nM (Table 1). The nature of the R moiety on the carboxamide functionality greatly influenced the inhibitory power. For hCA IX/XII the optimal substitution was that present in compound **15**, 2-trifluoromethylphenyl, whereas the one leading to the least effective inhibitor was the one with 4-bromophenylcarboxamide moiety (compound **9**) for hCA IX and 2-iodophenylcarboxamide (compound **12**) for hCA XII. Overall, all these new homosulfocoumarins act as isoform IX/XII selective CAIs over hCA I and II, which is highly desirable for these new chemotypes with enzyme inhibitory properties.

## 4. Conclusions

A series of 3H-1,2-benzoxathiepine 2,2-dioxides incorporating 7-acylamino moieties were obtained by an original procedure starting from 5-nitrosalicylaldehyde which was treated with propenylsulfonyl chloride followed by cyclisation through a Wittig reaction of the bis-olefin intermediate. The new derivatives, belonging to the homosulfocoumarin chemotype, were assayed as inhibitors of the zinc metalloenzyme CA. Four pharmacologically relevant human (h) isoforms were investigated, the cytosolic hCA I and II, and the transmembrane, tumour-associated hCA IX and XII. No relevant inhibition of hCA I and II was observed, whereas some of the new derivatives were effective, low nanomolar hCA IX/XII inhibitors, making them of interest for investigations in situations in which the activity of these isoforms is overexpressed, such as hypoxic tumours, arthritis or cerebral ischaemia.
